# Identification of a peptide-peptide binding motif in the coating of nab-paclitaxel nanoparticles with clinical antibodies: bevacizumab, rituximab, and trastuzumab

**DOI:** 10.1038/s41598-017-15251-6

**Published:** 2017-11-03

**Authors:** John T. Butterfield, Hidong Kim, Daniel J. Knauer, Wendy K. Nevala, Svetomir N. Markovic

**Affiliations:** 1 Division of Hematology, Mayo Clinic, Rochester, MN 55905 USA; 2 Division of Oncology, Mayo Clinic, Rochester, MN 55905 USA

## Abstract

Antibody directed chemotherapy (ADC) takes advantage of the selectivity of the monoclonal antibody to increase the efficacy of the chemotherapeutic agent, while reducing toxicity. Previously we described three nab-paclitaxel (Abraxane) nanoparticles coated with commercial monoclonal antibodies. Identifying the binding sites responsible for these particles could allow reverse engineering of nab-paclitaxel binding antibodies, creating a modular platform for antibody directed chemotherapeutic nanoparticles. Herein, Biacore surface plasmon resonance is used to identify an antibody binding site, HSA Peptide 40, on human serum albumin with nanomolar affinity for all three monoclonal antibodies. This 18-mer peptide, which lies in Subdomain IIIA of human serum albumin, blocks binding of all three antibodies to nab-paclitaxel when added in excess. We furthermore show the complementary binding region on all three monoclonal antibodies to be the CDR H3 loop of the Fab region, and show that they all have nano to micromolar affinity for HSA Peptide 40 and nab-paclitaxel nanoparticles. The presented data identify the nature of the critical protein-protein interaction that enables antibody coating of nab-paclitaxel.

## Introduction

Antibody drug conjugates (ADCs) present an opportunity to expand the usage of both monoclonal antibodies and chemotherapeutic drugs, while reducing the adverse effects of each. With the FDA approval of recent immune conjugates brentuximab (Adcetris) and T-DM1 (Kadcyla), as well as over 120 active clinical trials involving over 50 unique conjugates, ADCs are becoming an increasingly viable anticancer treatment^1^.

ADCs take advantage of the selectivity of the monoclonal antibodies to direct and deliver a highly cytotoxic chemotherapeutic agent to a tumor target. This has the potential to increase the drug efficacy by increasing the total delivery of toxic agent to tumor cells, while reducing non-specific toxicity. At the same time, ADCs provide an opportunity to re-purpose monoclonal antibodies that bind their tumor associated targets yet have little to no direct therapeutic effect, as well as repurpose cytotoxic agents that are too toxic (unacceptable side-effects) when delivered in non-directed fashion^2^.

We previously described an ADC platform of monoclonal antibodies non-specifically bound to paclitaxel containing human serum albumin (HSA) nanoparticles, nab-paclitaxel (Abraxane, ABX)^3^. ABX is a water soluble, 130-nanometer, nanoparticle of paclitaxel bound albumin that avoids the use of Cremaphor EL for paclitaxel infusion^4^. Cremophor has been associated with peripheral neuropathy as well as necessitating prolonged infusion times and antihistamine premedication^5^. We showed that the 130 nm ABX nanoparticles can be non-specifically bound and subsequently coated by the commercial monoclonal antibodies bevacizumab (anti-VEGF,Avastin), rituximab (anti-CD20, Rituxan), and trastuzumab (anti-HER2, Herceptin) to form 160-nm antibody/ABX nano-immunoconjugates (AB160, AR160, and AT160)^3^. This repurposing of humanized commercial antibodies avoids the high rates of immunogenicity of non-human antibodies used in most ADC’s^6,7^. After intravenous infusion the nano-immunoconjugate breaks into functional subunits containing albumin, paclitaxel, and the antibody^8^. These particles and the resulting functional units maintain the cytotoxicity of paclitaxel, as well as the ligand binding capability of the monoclonal antibody, resulting in increased *in vivo* efficacy due to improved tumor targeting^8^.

Characterizing the binding motif between the monoclonal antibody and the nab-paclitaxel nanoparticle could identify peptides with potential use as *in vivo* imaging probes as well as assisting in reverse engineering antibodies built to bind nab-paclitaxel nanoparticles, establishing a modular antibody directed chemotherapeutic platform. Previously, using Biacore Surface Plasmon Resonance (SPR) technology we identified an amino acid sequence on albumin (HSA Peptide 40, VVLNQLCELHEKTPVSDR) that bound the antibody rituximab with nanomolar affinity, and used a molar excess of the peptide to prevent formation of our AR160 nanoparticles, suggesting its role as the albumin-rituximab binding site in our monoclonal directed nanoparticles^8^. The similar affinities of rituximab, bevacizumab, and trastuzumab for nab-paclitaxel suggests their interaction is due to a similar binding site^3,8^. Herein, we show evidence to suggest that HSA Peptide 40 also serves as the binding site for bevacizumab and trastuzumab in our AB160 and AT160 nano-immunoconjugates, and identify the corresponding shared binding site between all three antibodies, for potential use in reverse engineered monoclonal antibodies.

## Results

### Identification of a Multiple Antibody Binding Peptide on Human Serum Albumin Using Biacore Surface Plasmon Resonance

**Figure 1:**
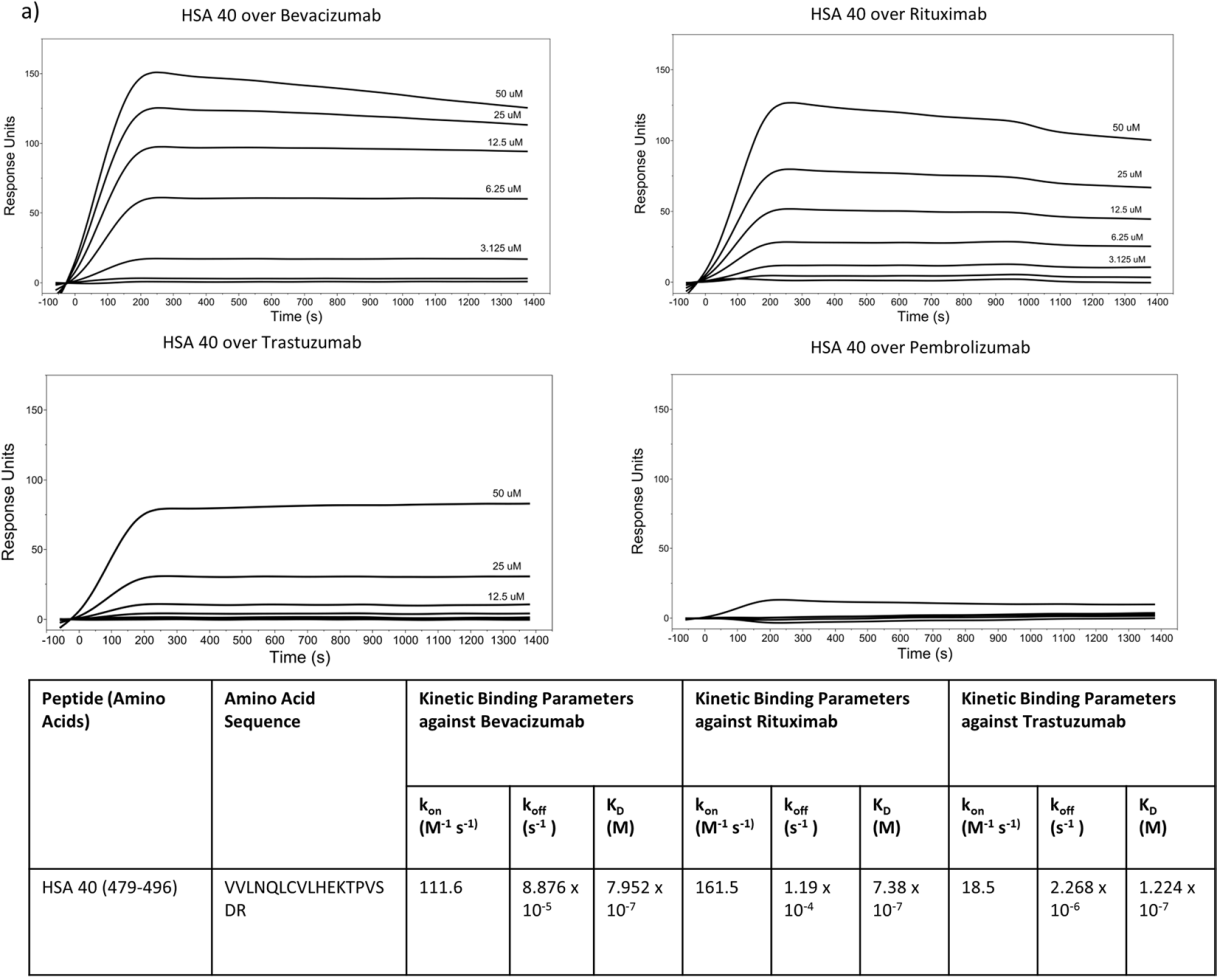
HSA Peptide 40 Binding to Monoclonal Antibodies Bevacizumab, Rituximab, and Trastuzumab Kinetic Binding Parameters Determined by Biacore-SPR: A peptide library of human serum albumin (HSA) was screened via Biacore over immobilized antibodies bevacizumab, rituximab, and trastuzumab. Antibodies were immobilized via amine coupling and peptides were run at pM-uM concentration ranges. (**a**) HSA Peptide 40 binding curves (180 second association period, 1200 second dissociation period) against all three antibodies and the negative control antibody pembrolizumab. (**b**) Binding kinetics were determined via Biacore Evaluation Software analysis of SPR sensograms.

We previously found nab-paclitaxel (Abraxane, ABX) can be bound and coated by the commercial antibodies bevacizumab (Avastin), rituximab (Rituxan), and trastuzumab (Herceptin) to form antibody directed chemotherapeutic nanoparticles^3,8^. To categorize the binding between the antibodies and albumin, a peptide library of human serum albumin (Supplementary Table 1) was ordered and screened against the three monoclonal antibodies using Biacore surface plasmon resonance (Fig. 1a). Three peptides were identified that bound at least one antibody, HSA peptide 4, HSA peptide 13, and HSA peptide 40. Out of those peptides only HSA peptide 40 bound all three antibodies, and not the negative control pembrolizumab. HSA 4 bound only rituximab and HSA 13 bound rituximab and bevacizumab but not trastuzumab, all with micromolar affinity. HSA Peptide 40 bound bevacizumab, rituximab, and trastuzumab with a binding affinity of 7.952 × 10^−7^, 7.38 × 10^−7^, and 1.224 × 10^−7^ molar, respectively (Fig. 1b). HSA peptide 40 has the amino acid sequence VVLNQLCVLHEKTPVSDR, corresponding to Val-445-Arg-472 in the HSA X-ray crystal structure, PDB accession code 1AO6.

**Figure 2:**
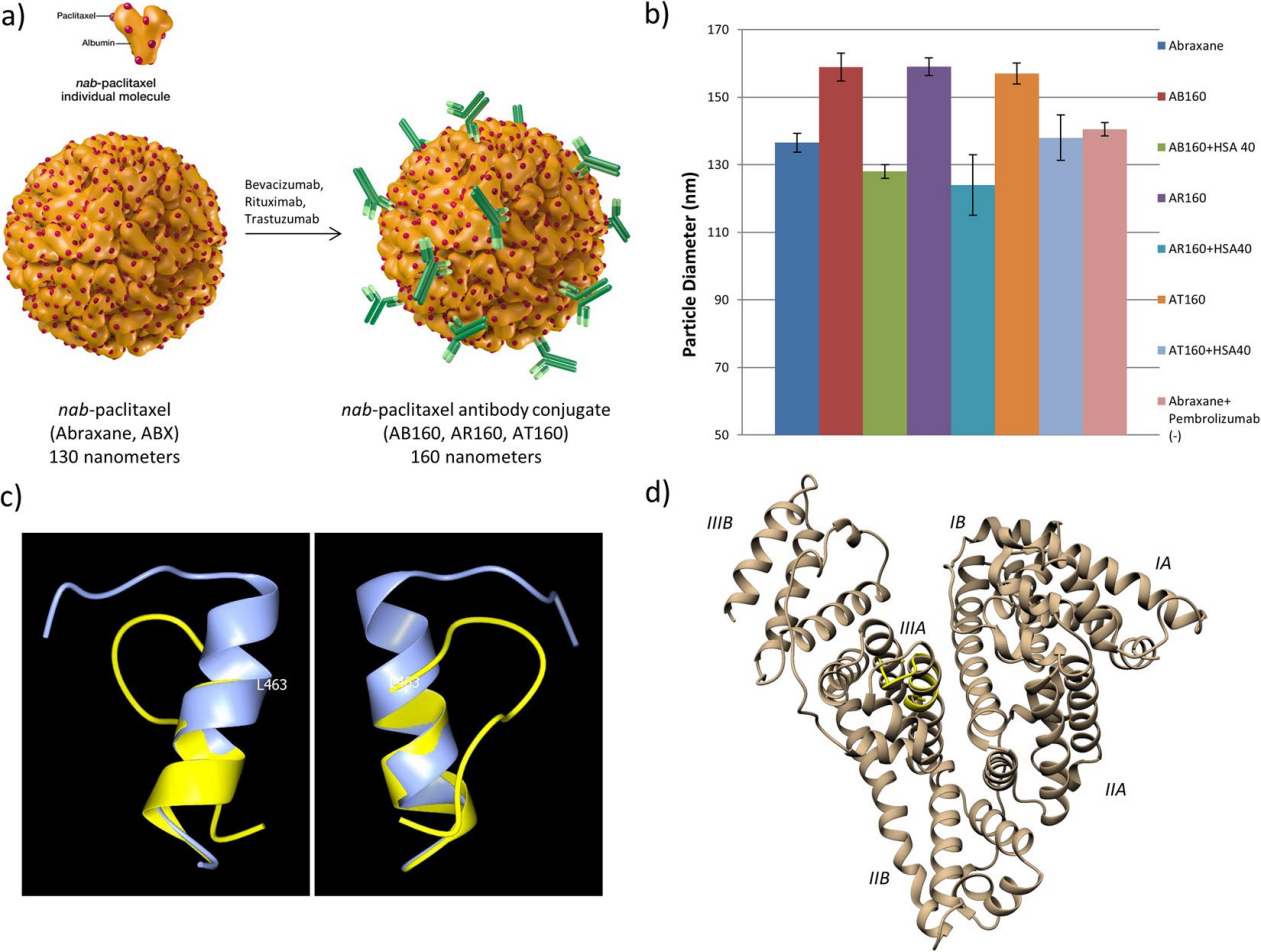
Monoclonal Antibody Coated Nab-Paclitaxel Nanoparticle Formation Inhibition by HSA Peptide 40. (**a**) Particle formation schematic, 130 nm nab-paclitaxel (Abraxane) nanoparticles are incubated with either bevacizumab (Avastin), rituximab (Rituxan), trastuzumab (Herceptin), or pembrolizumab (Keytruda) to form 160 nm antibody directed nanoparticles AB160, AR160, AT160, and a negative control Abraxane + Pembrolizumab. (**b**) 10 mg/ml of Abraxane was incubated for 30 minutes with 4 mg/ml of either bevacizumab, rituximab, or trastuzumab to test formation of AB160, AR160, or AT160 in the presence of a molar excess of HSA Peptide 40. Particle sizes were determined by NS300 dynamic light scattering and nanoparticle tracking system. Blocking of formation of 160 nm particles suggest HSA Peptide 40 outcompetes the in-particle albumin for the binding site on the antibodies. 3 repeats were performed for each experiment and error bars represent the standard error of measurement determined by Nanosight NTA 3.1 Software. (**c**) Superposition of predicted HSA peptide 40 model (yellow) onto Val-455 – Arg-472 in HAS X-ray crystal structure 1AO6 (blue). Structures drawn in ribbon representation. Two views approximately 180° apart. Leu-463 in 1AO6 labeled. (**d**) HSA Pep 40 (yellow) superimposed onto human serum albumin using MatchMaker, HSA domains and subdomains labeled.

To determine if HSA peptide 40 was indeed the site responsible for antibody binding in the nanoparticles AB160, AR160, and AT160, a molar excess of the peptide was added to the standard formulation as a blocking experiment. Incubation of 10 mg/ml of nab-paclitaxel (ABX) with 4 mg/ml of the monoclonal antibody in question causes an increase in particle diameter from 130-nm to 160-nm as the nab-paclitaxel nanoparticle is coated^3^ (Fig. 2a). A ten-fold molar excess of either HSA Peptide 40 or a non-binding control peptide was added to the standard formulation of AB160, AR160, and AT160. The diameter was measured by dynamic light scattering. Standard ABX nanoparticles measured 136.5 nm, AB160 measured 158.9 nm, AR160 measured 159 nm, and AT160 measured 157 nm. All three particles incubated with control peptides measured in the 157-160 nm range. When incubated with HSA Peptide 40, all three failed to form the 160 nm particle (Fig. 2b), measuring at 128, 124, and 138 nanometers (AB160, AR160, AT160). Pembrolizumab with Abraxane served as a negative antibody control, measuring 140.6 nm. A three dimensional model of HSA peptide 40 predicted by PEP-FOLD 3.0 shows the N-terminal portion in an alpha-helix and the C-terminal portion assuming a loop (Fig. 2c). On superposition of the predicted HSA peptide 40 model onto the corresponding Val-455 – Arg-472 portion of HSA structure 1AO6, the conformations of the two structures begin to diverge at Leu-463 of HSA 1AO6, with 1AO6 continuing as an alpha-helix (Fig. 2c)

### Biacore SPR Screening of a Peptide Library of Bevacizumab Identified a Fab Region Sequence Containing the CDR H3 Loop as the HSA Peptide 40 Binding Site

**Figure 3:**
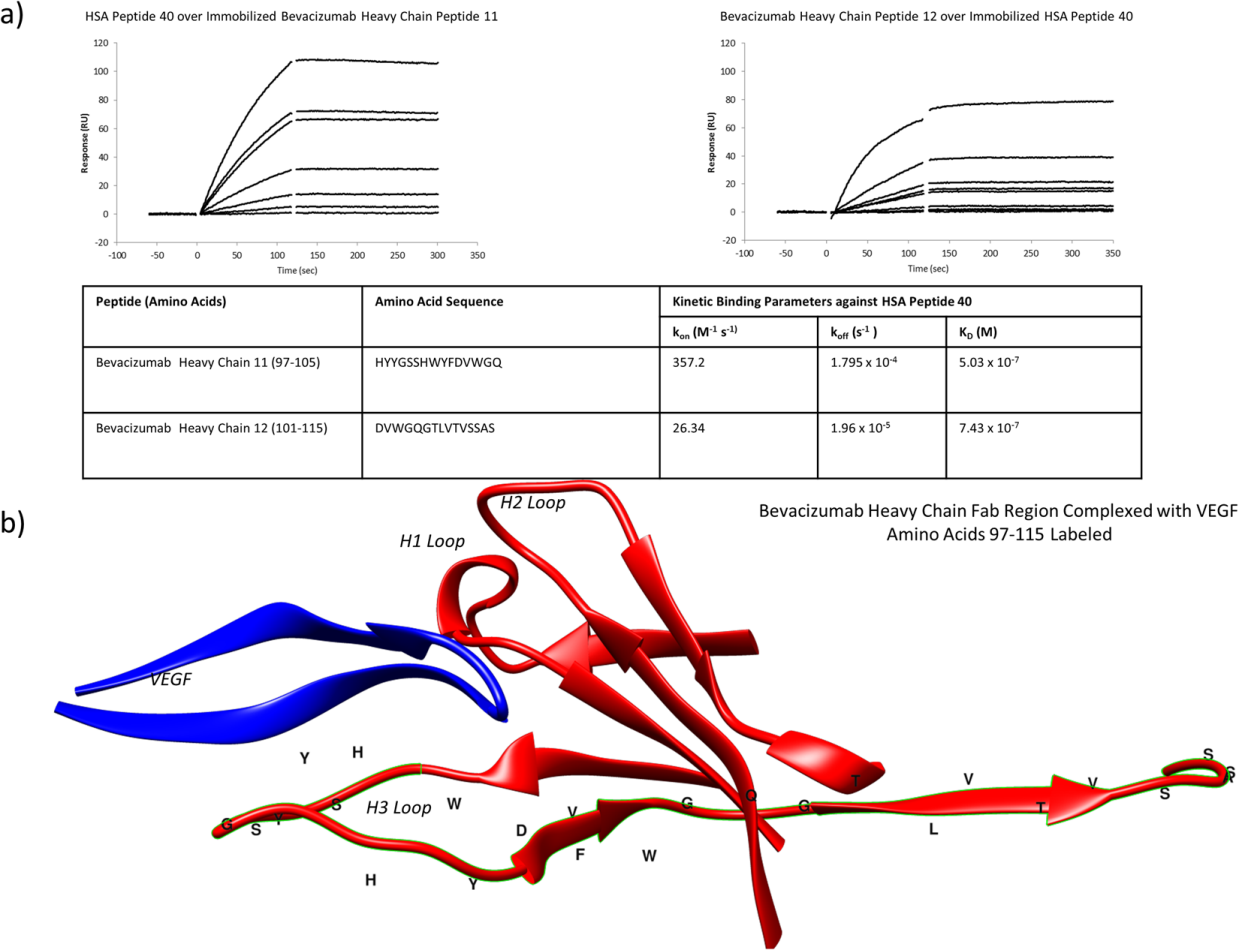
Bevacizumab Heavy Chain Peptide Library Kinetic Binding Parameters Determined by Biacore-SPR: A peptide library of bevacizumab was screened via Biacore SPR against HSA Peptide 40. The bevacizumab library was run over HSA Peptide 40 immobilized to a CM5 chip via amine coupling and vice versa. All peptides were run in the pM-uM concentration ranges. (**a**) Sensograms of HSA Peptide 40 run over immobilized Bevaciuzmab peptides 11 and 12 (120 second association period, 180 second dissociation period). Short gaps in binding curves at transition times are from removal of solvent spikes during analysis. Binding kinetics were determined via Biacore Evaluation Software analysis of SPR sensograms. (**b**) Location and structure of amino acids 90–122 on the heavy chain variable domain from a crystal structure of bevacizumab in complex with vascular endothelial growth factor (VEGF). Structure, PDB 1BJ1, was visualized using UCSF Chimera.

A peptide library of bevacizumab (Supplementary Table 2) was run over HSA peptide 40 immobilized on a Biacore CM5 chip. (Two concurrent peptides, and no others, bound HSA peptide 40, Bevacizumab peptides 11 and 12, which span the region of the antibody between amino acids 97–105 covering the sequence HYYGSSHWYFDVWGQGTLVTVSSAS (Fig. 3a). Bev 11 and 12 had K_a_’s of 357.2 and 26.34 (M^−1^s^−1^), K_d_’s of 1.795 × 10^−4^ and 1.96 × 10^−5^ (s^−1^), and binding affinities of 5.03 × 10^−7^ and 7.43 × 10^−7^ (M). This sequence was visualized using Chimera with a crystal structure of bevacizumab bound to vascular endothelial growth factor (PDB ID 1BJ1), and falls on the variable region of the heavy chain, encompassing the CDR H3 loop of the antigen binding site^9^ (Fig. 3b).

The same was repeated for a peptide library of rituximab (Supplementary Table 2) with no peptides showing affinity for HSA Peptide 40.

### The CDR H3 Containing Region is an ABX Binding Site Shared between Bevacizumab, Rituximab, and Trastuzumab

**Figure 4:**
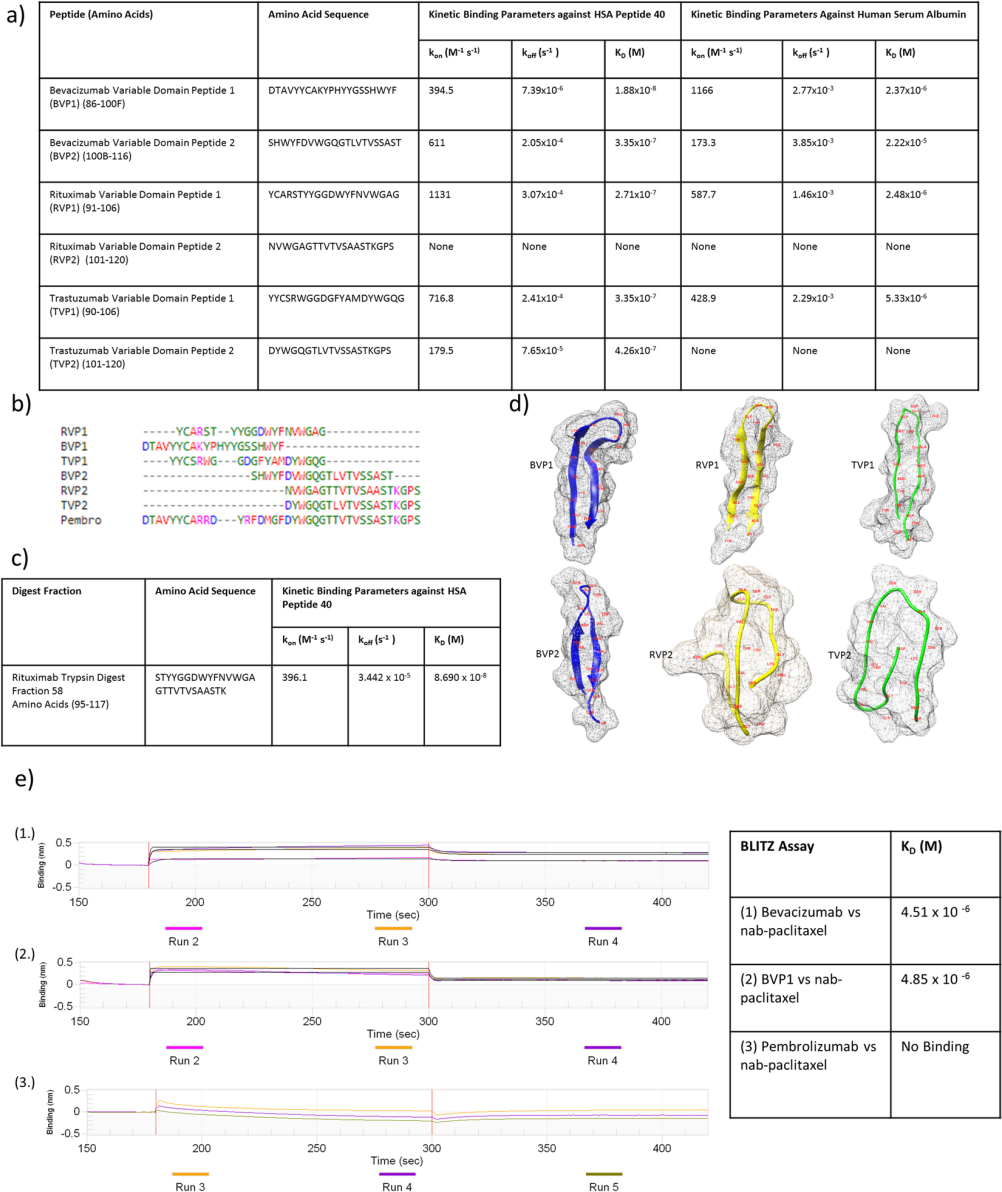
Bevacizumab, Rituximab, and Trastuzuzumab Heavy Chain Variable Domain Peptide Library Screening: A peptide library of the heavy chain variable domain for Bevacizumab, Rituximab, and Trastuzumab were screened via Biacore over immobilized HSA and an HSA peptide library, as well as vice versa. Peptides were run in the pM-uM concentration range. (**a**) The positive results of the peptide screening. Binding kinetics between antibody variable domain peptides and HSA Peptide 40/HSA were determined by Biacore Evaluation Software analysis of SPR sensograms. (**b**) Multiple sequence alignment of the variable region peptides in question, performed by Clustal Omega. (**c**) Rituximab digest Biacore screening. Rituximab was trypsin digested and separated by HPLC reverse phase chromatography. The fraction (Fraction 58) that bound both HSA Peptide 40 and albumin was sequenced via mass spec. (**d**) 3D structure prediction of peptides in solution performed by PEP-FOLD 3.0, visualized in Chimera. (**e**) Biolayer Interfermoetry (BLITZ) assay kinetics assay of either biotinylated bevacizumab or Bev Variable Domain Peptide bound to strepdavidin probe against Abraxane nanoparticles. Pembrolizumab included as a negative control.

After the identification of bevacizumab peptides 11 and 12, a peptide library was synthesized of the same region in rituximab, trastuzumab, and repeated in bevacizumab, with extensions in both N and C terminal directions (Supplementary Table 3). These heavy chain variable domain libraries were tested via Biacore over immobilized HSA and HSA Peptide 40. Then each variable domain peptide was immobilized and had HSA or HSA peptide 40 run over it (Fig. 4a). Bevacizmuab Variable Domain Peptides (BVP) 1 and 2 bound both analytes while BVP3 bound neither. Rituximab Variable Domain Peptide 1 (RVP1) bound both with RVP2 and 3 binding neither. Trastuzumab Variable Domain Peptide 1 (TVP1) bound both analytes, TVP2 bound HSA peptide 40 but not albumin, TVP3 bound neither. All HSA 40 positive peptides showed overlap spanning the region of amino acids that make up the H3 loop of the complementarity determining region (CDR) (Fig. 4b), while those that split the loop (RVP2, TVP2) did not bind the full HSA. To further reinforce the Fab region H3 loop as the binding site, rituximab was digested by trypsin and separated using reverse-phase chromatography. Theses fractions were screened over immobilized HSA peptide 40 and the positive result was reported and sent to Mayo Clinic Medical Genome Facility Proteomics Core for sequencing by mass spectrometry (Fig. 4c), returning a sequence covering amino acids 95–117. The 3D structure of the positive variable region peptides were predicted by PEPFOLD 3.0 to compare to their beta hairpin structure within the full antibody^9–11^ (Fig. 4d). Because a full ABX nanoparticle does not remain stable at the concentrations required for Biacore testing^12^, BVP1 was biontinylated, bound to a streptavidin probe, and tested via Bio layer interferometry (BLITZ) for ABX nanoparticle affinity (Fig. 4e). Resulting in a K_D_ of 4.85 × 10^−6^, matching that of the full antibody versus nab-paclitaxel, 4.51 × 10^−6^.

## Discussion

Nab-Paclitaxel nanoparticles can be non-specifically coated with the monoclonal antibodies rituximab, bevacziumab, and trastuzumab, to form antibody drug conjugates^3,8^. These ADC’s showed increased *in vivo* efficacy due to antibody tumor targeting. ADC’s are clinically pressing because they can increase the efficacy of a chemotherapeutic agent by delivering a higher percentage to the target cell, using antigen targeting antibodies^2^. This allows a clinician to give higher doses as the off-site toxicity is reduced. Rituximab conjugated nab-paclitaxel is currently in Phase 1 clinical trials in treatment of CD20+ Lymphoma, as is bevacizumab coated nab-paclitaxel for Melanoma.

Discovering the binding sites between the antibodies and albumin would elucidate the oddity of three different antibodies sharing multispecificity for the same molecule. The similarities in affinity of all three antibodies for nab-paclitaxel suggest the interaction of a common binding site^3,8^. This shared motif between the monoclonals could be used to reverse engineer other antibodies and make ADC nab-paclitaxel nanoparticles for many antigen targets. It would also allow for repurposing of past antibodies with strong kinetic properties but poor therapeutic value^2^.

Through surface plasmon resonance we identified only one sequence in an overlapping 18-mer human serum albumin peptide library that bound all three antibodies. HSA peptide 40 (VVLNQLCVHEKTPVSDR) had affinity for all three antibodies in the nanomolar range (Fig. 1b). The strength of this binding motif comes from dissociation rates in the micromolar range, despite association rates in the hundreds. HSA peptides 4 and 13, were not further investigated as they did not bind all three antibodies, therefore could not describe the similar binding affinity of each antibody for nab-paclitaxel. Furthermore, their affinities in the micromolar range were less than the antibody-particle affinity in the nanomolar range, suggesting HSA 4 and 13 results may be due to nonspecific hydrophobic interactions with albumin.

Further supporting HSA 40 as the responsible antibody binding site, and not a non-specific binding peptide, a molar excess of the peptide added to our normal particle incubation process prevented formation of the 160 nanometer particles, the typical size of antibody coated nab-paclitaxel, for all three antibodies (Fig. 2b). These results suggest that HSA Peptide 40 bound the antibodies, occupying their corresponding binding site, and inhibited them from binding the albumin in nab-paclitaxel. Because the nanosight technology reads the average nanoparticle size, there is the possibility that a small percentage of the antibody bind through other interactions that would cause too small of a diameter change to see with limited sensitivity. The peptide in solution and in the full HSA molecule share some structural elements, an N-terminal alpha helix (toward the core of HSA) followed by a short loop (at the protein surface) which begins tracking back toward the core^13,14^.

Superposition of HSA Peptide 40 onto albumin (Fig. 2d) shows the peptide’s location in Domain IIIA of the full molecule, directly adjacent to the hydrophobic drug-binding site, Sudlow’s Site II^15^. Its relatively interior location is not typical of an antibody binding site, and would be expected to encounter steric hindrance with the antibody protein^16^. Residues Val-455 – Arg-472 of HSA structure 1AO6 have relatively little solvent accessible surface area for the N-terminal residues. There is greater solvent accessible surface area for the C-terminal residues which are closer to the surface of 1AO6 (supplemental Table 4). The poor solvent accessibility of much of Val-455 – Arg-472 in HSA structure 1AO6 combined with the observation that bevacizumab, rituximab, and trastuzumab do not bind HSA unless in the nab-paclitaxel particle form, suggests that the HSA in nab-paclitaxel in likely varies conformationally from that typically found in serum. Discussion of the BLItz data below will address how the pressure-based processing of HSA in the nab-paclitaxel formulation may play a role in exposing this HSA 40 binding site. Never the less, because of its high affinity for multiple commercially available antibodies, HSA Peptide 40 presents an opportunity for use as a universal antibody linker molecule, amendable to a number of pre-clinical and clinical diagnostic and therapeutic appliactions^17^.

Screening of a bevacizumab peptide library over HSA Peptide 40 revealed a region with 10^−7^ M affinity for HSA Peptide 40, covered by the peptides Bevacizumab Heavy Chain 11 and 12 (Fig. 3a). This region contains residues 97–115 of the heavy chain of the antibody bevacizumab. Screening of a rituximab heavy chain library showed no peptides that bound HSA 40. This is believed to be due to the same region in rituximab being split between two peptides in this specific heavy chain library. This is further supported later by the high affinity of RVP1 for HSA 40, as RVP1 dose not as drastically split the rituximab region of interest.

These residues in bevacizumab make up part of the anti-VEGF Fab region, specifically the CDR H3 loop, which spans Tyr95-Val102 (Fig. 3b). When in complex with VEGF, four CDR loops are in contact with the antigen. H3 is the most significant as it is responsible for 50% of the buried surface area^13^. These results suggest that bevacizumab binds HSA, and by extension the entire nab-paclitaxel nanoparticle, via its Fab region, raising concerns of the effect on the antibody-antigen interaction. Despite potential hindrance, we have previously shown via flow cytometry that AB160 and AR160 both maintain their ability to bind their antigens, VEGF and CD20, respectively^3,8^. One possible explanation is that the second Fab region remains unbound and free to associate with the antigen.

Peptides encompassing the H3 loop of rituximab and trastuzumab were also found to bind HSA Peptide 40 and albumin (Fig. 4a). This shared albumin binding site is a potential explanation for all three antibodies (bevacizumab, rituximab, trastuzumab) sharing multispecificity for nab-paclitaxel nanoparticles. Identification of the activity of this H3 loop and the shared amino acids allow us to screen a wide range of antibodies by sequence and structure to identify possible candidates for future use in antibody directed nab-paclitaxel particles, with the possibility of reverse engineering this structural motif into clinically relevant antibodies as well. For each H3 encompassing antibody library no peptides beginning beyond amino acid 111 showed any binding activity. While the most overlap occurred between amino acids 100 and 106-7, encompassing the letter affiliated 100a-f amino acids typical of the Kabat numbering sequence (Fig. 4b). Of the peptides sharing that overlap, BVP1, BVP2,RVP1, and TVP1 all bound both HSA and HSA peptide 40 with nano to micromolar affinity. The variation in affinity between the peptides of the three antibodies is also notable, suggesting that the antibodies themselves may have structural or amino acid sequence variations that cause the reported differences in their affinity for ABX^3,8^.

Without a crystal structure of HSA peptide 40 bound to all four antibodies, it is difficult to determine the exact amino acids responsible on the CDR H3 loop. With publically available sequences and structures for the antibodies a comparison can be drawn for further investigation, though it is still subject to speculation. The CDR H3 loop on the negative control Pembrolizumab has bulky phenylalanine and arginine as the most superficial residues on the loop, with their side chains protruding away from the protein core (Supplementary Figure 1), while bevacizumab, rituximab, and trastuzumab have glycine residues in the more superficial locations. These glycine residues at the peptide turn may not have the steric hindrance present in pembrolizumab and add chain flexibility, allowing HSA 40 to bind. It’s worth noting that trastuzumab also has a phenylalanine near the loop turn, but the side chain is directed into the core of the protein. This could potentially account for the ten times decrease in association rate seen of HSA 40 to trastuzumab seen in Fig. 1. Screening of antibodies for future use with nab-paclitaxel or HSA 40 could be done, superficially, by analyzing the amino acid sequence at the turn of the CDR H3 loop and looking for favorable non-bulky residues and avoiding unfavorable bulky, hydrophobic residues.

In comparison to the four peptides that bound both analytes, TVP2 bound only HSA Peptide 40, and RVP2 bound neither, despite some overlap with the others (Fig. 4b). One possible explanation for this is that both of these peptides begin at amino acid 101, while the other four double positive peptides contain all the lettered 100 amino acids (100a-100f for bevacizumab, 100a-100d for rituximab, 100a-100c for trastuzumab). This is further reinforced by the fact that the sequence of pembrolizumab, which does not bind nab-paclitaxel, shares identical sequence to trastzuzumab post amino acid 101 but varies significantly within the kabat lettered amino acid section in comparison to all three positive antibodies. Another aspect for further investigation is the variation in predicted 3d structure of RVP2 and TVP2 versus the four double positive peptides (Fig. 4d). BVP1-2, RVP1, and TVP1 all have a single beta hairpin structure, representative of the H3 loop found within the antibodies themselves. RVP2 and TVP2 on the other hand have a stretch of amino acids that circle back for a second loop, a stretch that is normally tucked into the core of the antibody^9,18,19^. This second looping could cause steric hindrance that prevents HSA binding.

A trypsin digest of the full rituximab antibody revealed a region that bound HSA peptide 40 with nanomolar affinity (Fig. 4c). After sequencing, this fraction was determined to contain amino acids 95–117 on the heavy chain. As a separate method for identification, this provides strong support for the region previously discovered through a peptide library. Amino acids 95–117 represents the largest stretch of amino acids shown to bind HSA Peptide 40, and is therefore the sequence most likely to accurately represent the full three-dimensional structure of this region within the full antibody^20^.

We have shown that ABX nanoparticles can be bound by rituximab, bevacizumab, and trastuzumab^3^, and that these three antibodies bind HSA Peptide 40 through the CDR H3 loop of their Fab regions. If this CDR H3-HSA peptide 40 binding motif is responsible for the antibodies binding ABX then the CDR H3 region should also show affinity for the nab-paclitaxel particle as a whole. Because nab-paclitaxel is both too large and too unstable for use in Biacore^12^, we used Bio Layer Interferometry (BLItz). BVP1 and bevacizumab were both biotinylated, attached to a streptavidin probe, and assayed against nab-paclitaxel. Their affinities for nab-paclitaxel were nearly identical at a K_D_ of 4.85 × 10^−6^ for BVP1 and 4.51 × 10^−6^ for bevacizumab, further supporting the CDR H3-HSA Peptide 40 binding motifs role in the antibody bound and directed nab-paclitaxel particles.

Future research is needed in determining how the antibodies bind HSA Peptide 40. The fact that this peptide is located relatively interiorly within the full HSA molecule and adjacent to a very hydrophobic drug binding site^15^, paired with the lack of binding between the full antibodies and albumin from sources other than nab-paclitaxel particles, suggests that something in the process of nab-paclitaxel formation permits antibody-albumin binding that otherwise would not happen. Previously, Tanaka *et al*. have shown that Domain III of HSA, containing HSA Peptide 40, is particularly susceptible to pressure induced unfolding and have even used pressure to modify buried amino acid residues^21,22^. Because the formulation of nab-paclitaxel includes high-pressure homogenization up to 30,000 PSI^23^, further research could pursue whether pressure induced structural changes make the region in HSA corresponding to HSA peptide 40 more accessible for antibody binding.

Conventional methods of forming ADC’s involve using a synthetic linker to covalently bind the cyotoxic agent to the antibody. These linkers conjugate at exposed lysine or cysteine residues on the antibody, an unspecific approach that leads to many different ADC species with distinct pharmacokinetics to address. This is the case with clinically available ADC’s brentuximab vedotin (Adcetris) and ado-trastuzumab emtansine (Kadcyla)^2^. Our nano-immunoconguate platform uses a non-covalent peptide-peptide binding motif that simplifies the process of conjugation. Because of the clinical availability of ABX and the identification of the binding motif on the antibody, this platform presents an opportunity to address and develop ADC’s using an expanding array of clinically relevant monoclonal antibodies.

## Materials and Methods

### Peptide Libraries

A peptide library of human serum albumin was made of 18 amino acid peptides with a 5 amino acid overlap (Supplementary Table 1). A peptide library for the heavy chain of bevacizumab was made of 14 amino acid peptides with a 4 amino acid overlap (Supplementary Table 2). A peptide library for the heavy chain of rituximab was made in the same manner (Supplementary Table 2). After identification of an area of interest, three more peptide libraries were ordered of the heavy chain variable region of three nab-paclitaxel binding antibodies; bevacizumab, rituximab, and trastuzumab (Supplementary Table 3). This library consisted of 19–20 amino acid peptides with significant overlap. All peptides were provided by New England Peptide (Gardner, MA, USA), and numbered following the Kabat numbering scheme^24^.

### Antibody and Peptide Binding Assay via Surface Plasmon Resonance

Peptides were re-suspended and stored in HBS running buffer at either 5 or 10 mg/ml, with up to 10% DMSO added as needed. All antibodies, peptides, as well as HSA, were immobilized on Biacore (GE Healthcare, Chicago, IL) CM5 chips via amine coupling according to manufacture protocol. Immobilization scouting was performed with pH 4.0–5.5 acetate at micromolar concentration ranges, to determine ideal immobilization conditions. Biacore X-100’s were used to screen the monoclonal antibodies and their heavy chain libraries against immobilized HSA and HSA peptide 40. All peptides and proteins were screened at multiple concentrations from pico to millimolarity, with an association time of 120 to 180 seconds and a dissociation time from 180 to 1200 seconds. Binding kinetics were determined using Biacore Evaluation Software and a 1:1 fit model.

### Nab-Paclitaxel and Antibody Conjugate Nanoparticle Formation with HSA Peptide 40 Competition

10 mg/ml of nab-paclitaxel dose, 100 mg/ml total mass, was incubated for 30 minutes with 4 mg/ml of the antibody in question and a 10 times molar excess of the peptides in question. After incubation, nanoparticle diameter was measured using Dynamic Light Scattering Nanoparticle Tracking Analysis via a Malvern Nanosight (Malvern, Worcestshire, UK). Pembrolizumab, as a non-abraxane binding antibody, was used as a negative control along with Abraxane. Measurements were made at a 1:200 dilution, with 5 60 second captures at a syringe pump flow rate of 25 (arbitrary units). Incubations were repeated 3 times; measurements were batched and analyzed using the Malvern NTA software.

### Three-Dimensional Protein Visualization and Peptide Structure Prediction

Crystal structures of both bevacizumab in complex with vascular endothelial growth factor (PDB 1BJ1), as well as human serum albumin (PDB 1AO6) were obtained from RCSB Protein Data Bank and visualized by UCSF Chimera^24^. Three-dimensional peptide structures were predicted by PEP_FOLD 3.0^10,11^. Superpostioning of peptides was done using Chimera MatchMaker tool using the Needleman-Wunsch alignment algorithm, and LSQKAB from the CCP4 suite^25,26^.

### Rituximab Digest, separation, binding analysis, and identification

Rituximab was purified using a Thermo Fisher Protein A (Thermo Fisher Scientific, Waltham, MA) column according to manufacture protocol, run through a desalting column, and lyophilized overnight. Rituximab and trypsin (ThermoFisher Cat # 90057) were incubated overnight at a 1:20 w/w ratio in a 500 mM Ammonium Bicarbonate solution. The mixture was frozen, evaporated, and re-suspended in HPLC grade water with 0.1% trifluoroacetic acid. The digest was applied to a Waters X-Bridge Protein BEH C4 column (Waters, Milford, MA) and eluted off at a flow rate of 1 ml/min and a gradient of 0–60% Acetonitrile with 0.1% trifluroacetic acid over 60 minutes. 1 minute fractions were collected, lyophilized, and re-suspended in 100 ul of HBS for use in Biacore binding assays against albumin and HSA Peptide 40.

### Bio Layer Interferometry Kinetic Assay (BLItz)

Bevacizumab Variable Domain Peptide 1 was biotinylated using a Thermofisher EZ Link NHS-PEO4-Biotinylation Kit. To avoid the biotin label from hindering BVP1-nab paclitaxel binding, BVP1 was preferentially labeled at the N-terminal by lowering the pH to 6.5, using a biotin to peptide molar ratio of 1 to 5, and incubating at 4 °C for 24 hours. After incubation, excess biotin was removed using a PD Midi-Trap G10 from GE Life Sciences. A BLItz advanced kinetics (ForteBio Corp. Menlo Park, CA) assay was run, streptavidin probes were incubated with 50 ug/ml biotinylated BVP1. Nab-paclitaxel or albumin controls were probed at multiple concentrations in the nano-millimolar range. Kinetic constants were calculated by the BLItz analysis software.

### Data Availability

All data generated during and/or analyzed during the current study are available from the corresponding author upon reasonable request.

## Electronic supplementary material

Supplementary Figures
