# Supplementary material for: Identification of a peptide-peptide binding motif in the coating of nab-paclitaxel nanoparticles with clinical antibodies: bevacizumab, rituximab, and trastuzumab
Source: Sci Rep. 2017 Nov 3;7:14476. doi: 10.1038/s41598-017-15251-6 (PMC5670201; doi:10.1038/s41598-017-15251-6)
Supplement: Supplementary file 1 — Supplementary Figures [file 41598_2017_15251_MOESM1_ESM.doc]

**Supplementary Figures**

**Identification of a peptide-peptide binding motif in the coating of nab-paclitaxel nanoparticles with clinical antibodies: bevacizumab, rituximab, and trastuzumab**

John T. Butterfield^1^, Hidong Kim^1,2^, Daniel J. Knauer^1^, Wendy K. Nevala^1^, and Svetomir N. Markovic^1,2^*

^1^Division of Hematology, Mayo Clinic, Rochester, MN 55905

^2^Division of Oncology, Mayo Clinic, Rochester, MN 55905

^*^markovic.svetomir@mayo.edu

**Supplementary Table 1: Human Serum Albumin Peptide Library:** HSA sequence numbered according to UnitProtKB, accession number P02768, including signal and propeptide.

|  | **Rituximab Heavy Chain** | |  |  |
| --- | --- | --- | --- | --- |
| 1 |  |  | QVQLQQPGAELVKPGASV | |
| 2 |  |  | KPGASVKMSCKASGYTFT | |
| 3 |  |  | GYTFTSYNMHWVKQTPGR | |
| 4 |  |  | QTPGRGLEWIGAIYPGNG | |
| 5 |  |  | YPGNGDTSYNQKFKGKAT | |
| 6 |  |  | KGKATLTADKSSSTAYMQ | |
| 7 |  |  | TAYMQLSSLTSEDSAVYY | |
| 8 |  |  | SAVYYCARSTYYGGDWYF | |
| 9 |  |  | GDWYFNVWGAGTTVTVSA | |
| 10 |  |  | VTVSAASTKGPSVFPLAP | |
| 11 |  |  | FPLAPSSKSTSGGTAALG | |
| 12 |  |  | TAALGCLVKDYFPEPVTV | |
| 13 |  |  | EPVTVSWNSGALTSGVHT | |
| 14 |  |  | SGVHTSGVHTFPAVLQSS | |
| 15 |  |  | VLQSSGLYSLSSVVTVPS | |
| 16 |  |  | VTVPSSSLGTQTYICNVN | |
| 17 |  |  | ICNVNHKPSNTKVDKKAE | |
| 18 |  |  | DKKAEPKSCDKTHTCPPC | |
| 19 |  |  | TCPPCPAPELLGGPSVFL | |
| 20 |  |  | FPPKPKDTLMISRTPEVT | |
| 21 |  |  | TPEVTCVVVDVSHEDPEV | |
| 22 |  |  | EDPEVKFNWYVDGVEVHN | |
| 23 |  |  | VEVHNAKTKPREEQYNST | |
| 24 |  |  | EQYNSTYRVVSVLTVLHQ | |
| 25 |  |  | LTVLHQDWLNGKEYKCKV | |
| 26 |  |  | YKCKVSNKALPAPIEKTI | |
| 27 |  |  | IEKTISKAKGQPREPQVY | |
| 28 |  |  | EPQVYTLPPSRDELTKNQ | |
| 29 |  |  | LTKNQVSLTCLVKGFYPS | |
| 30 |  |  | GFYPSDIAVEWESNGQPE | |
| 31 |  |  | NGQPENNYKTTPPVLDSD | |
| 32 |  |  | VLDSDGSFFLYSKLTVDK | |
| 33 |  |  | KLTVDKSRWQQGNVFSCS | |
| 34 |  |  | VFSCSVMHEALHNHYTQK | |
| 35 |  |  | HYTQKSLSLSPGK | |

**Supplementary Table 2: Bevacizumab and Rituximab Heavy Chain Peptide Libraries**

**Supplementary Table 3: Bevacizumab, Rituximab, Trastuzumab Extended Variable Region Peptide Library**

**
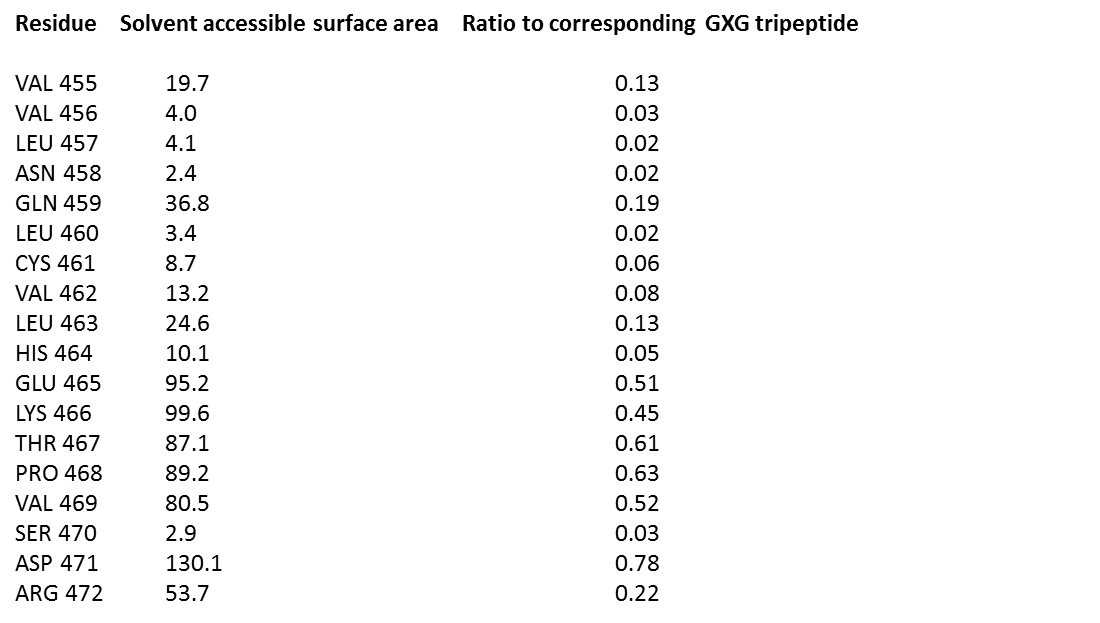
**

**Supplementary Table 4. Solvent accessible surface area for residues Val-455 – Arg-472 in HSA crystal structure 1AO6.** Solvent accessible surface area calculated using 1.4-Å radius solvent probe in AREAIMOL. For each residue, solvent accessible surface area in Å^2^, and ratio of solvent accessible surface area to that of corresponding GXG tripeptide shown.


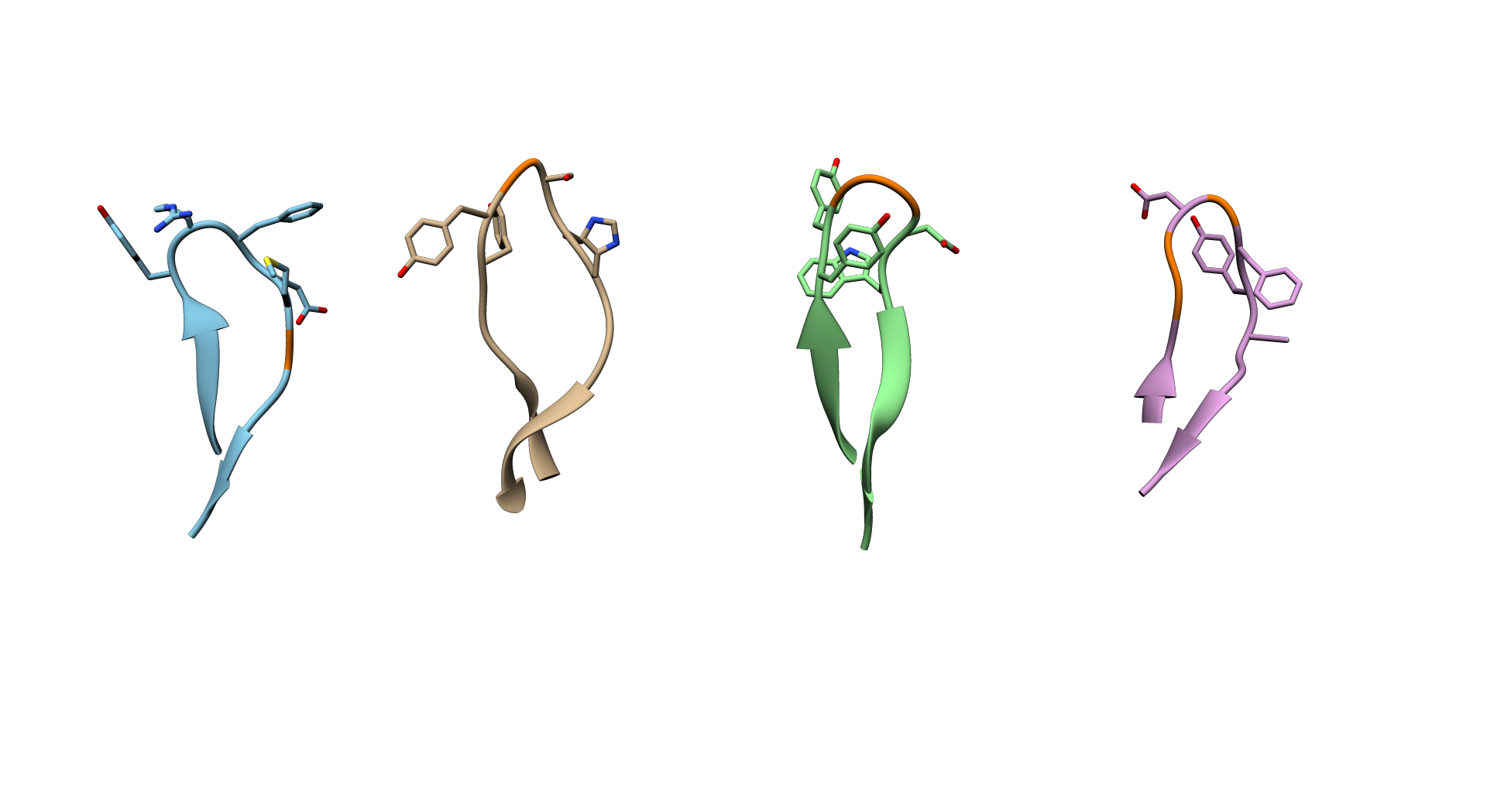


**Pembrolizumab**

**Bevacizumab**

**Rituximab**

**Trastuzumab**

**Supplementary Figure 1. CDR H3 Loop of Pembrolizumab, Bevacizumab, Rituximab, and Trastuzumab from PDB Crystal Structures.** All H3 loops displayed from 3d Structures PDB ID 5GGS, 1BJ1, 4KAQ, and 1N8Z. Glycine residues are marked in orange.
